# Fast-TrACC: A Rapid Method for Delivering and Testing Gene Editing Reagents in Somatic Plant Cells

**DOI:** 10.3389/fgeed.2020.621710

**Published:** 2021-01-20

**Authors:** Ryan A. Nasti, Matthew H. Zinselmeier, Macy Vollbrecht, Michael F. Maher, Daniel F. Voytas

**Affiliations:** 1 Department of Genetics, Cell Biology and Development, University of Minnesota, St. Paul, MN, United States; 2 Center for Precision Plant Genomics, University of Minnesota, St. Paul, MN, United States; 3 Center for Genome Engineering, University of Minnesota, St. Paul, MN, United States; 4 Plant and Microbial Biology Graduate Program, University of Minnesota, St. Paul, MN, United States

**Keywords:** CRISPR, gene editing, plant, solanacaeae, *Agrobacaterium tumefaciens*, reporter

## Abstract

The production of transgenic or gene edited plants requires considerable time and effort. It is of value to know at the onset of a project whether the transgenes or gene editing reagents are functioning as predicted. To test molecular reagents transiently, we implemented an improved, *Agrobacterium tumefaciens*-based co-culture method called Fast-TrACC (Fast Treated Agrobacterium Co-Culture). Fast-TrACC delivers reagents to seedlings, allowing high throughput, and uses a luciferase reporter to monitor and calibrate the efficiency of reagent delivery. We demonstrate the use of Fast-TrACC in multiple solanaceous species and apply the method to test promoter activity and the effectiveness of gene editing reagents.

## INTRODUCTION

Producing a gene edited plant requires considerable time, often from 6 to 9 months (Altpeter et al., 2016). Over this time period, significant effort must be put forth to identify edited cells in culture and induce them to form shoots and roots. Because of this investment in time and labor, it is important to know at the onset of an experiment whether the gene editing reagents can effectively create the desired genetic change. Typically, reagents are tested using transient assays to determine reagent efficacy within a shorter timescale. By comparing several different reagents in this manner the most efficient one can be selected and used to generate the gene edited plant. Currently, the most common transient delivery systems involve protoplasts (Lin et al., 2018) or leaf infiltrations (Janssen and Gardner, 1990; Ali et al., 2018). While both are effective, each has its own associated drawbacks. Protoplast isolation, where one removes the cell wall from plant cells, allows for transient transformation by chemical methods or electroporation. Isolating protoplasts is technically challenging and places the cells in an unnatural environment. On the other hand, leaf infiltration, performed by perfusion of *Agrobacterium tumefaciens* into a leaf with a needless syringe, is simple to perform but works with a limited number of plants, and time is required to grow plants to the proper stage for infiltration.

An alternative method, called AGROBEST, was developed for transient expression of transgenes in *Arabidopsis thaliana* (Wu et al., 2014). In this method Agrobacterium cultures are placed in media to promote expression of the *vir* genes, thereby improving the efficiency of T-DNA transfer to plant cells. With this increase in *vir* expression, one can deliver a given T-DNA cargo by simply co-culturing Arabidopsis seedlings with the treated bacterial culture. We sought to use this approach to deliver T-DNA cargo to *Nicotiana benthamiana* seedlings, however, in order to achieve transformation, it was necessary to make changes to the concentration of bacteria used and the length of time the seedlings and bacteria were co-cultured (Maher et al., 2020). Specifically, increasing the Agrobacterium concentration and shortening co-culture times resulted in improvements in transgene delivery. This altered method, fast treated Agrobacterium co-culture (Fast-TrACC), was used to deliver developmental regulators to *N. benthamiana* seedlings to induce *de novo* meristems to create either transgenic or gene edited shoots (Maher et al., 2020).

The success of Fast-TrACC in *N. benthamiana* suggested that it might be generally useful as a transient DNA delivery method. Here we show success in using Fast-TrACC to efficiently deliver transgenes to other related species, including tomato (*Solanum lycopersicum*), potato (*Solanum tuberosum*), pepper (*Capsicum chinense*), and eggplant (*Solanum melongena*). We also used Fast-TrACC to compare the activity of various promoters in these species using a luciferase reporter, and we demonstrate that Fast-TrACC can quickly assess the activity of gene editing reagents at endogenous chromosomal targets. With relative ease, Fast-TrACC makes it possible to identify the reagents with highest activity prior to generating a gene edited plant line.

## METHODS

### DNA Constructs

All constructs generated for the Fast-TrACC experiments (Supplementary Table 1) were cloned into a T-DNA backbone to allow for Agrobacterium-mediated gene transfer. The majority of cloned T-DNA backbone includes sequence elements that produce Bean Yellow Dwarf Virus (BeYDV) or Tomato Leaf Curl Virus (ToLCV) geminiviral replicons, which circularize and replicate (Baltes et al., 2014). Replication increases copy number of the vector and consequently leads to high levels of gene expression. Whereas, replicons provide increased expression, they are not required, as non-replicon T-DNAs were used for the dual luciferase promoter comparison assay. Construct assembly was performed via a modular Golden Gate cloning platform (Čermák et al., 2017).

Two types of constructs were used in the Fast-TrACC experiments: luciferase reporter constructs and gene editing constructs. The reporter constructs were intended to express either firefly or Renilla luciferase (Thorne et al., 2010) using various promoters. Two types of promoters were tested: (1) strong promoters like cauliflower mosaic virus *35S* or Arabidopsis *Ubiquitin 10* (*AtUbi10*); (2) promoters with variable (Cestrum Yellow Leaf Curling Virus, *CmYLCV*) or undefined expression levels (Arabidopsis ribulose-1,5-bisphosphate carboxylase/oxygenase small subunit, *AtRbcs*) (Engler et al., 2014; (Čermák et al., 2017). The gene editing T-DNA vectors were designed to express the RNA guided endonuclease, SpCas9, driven by the *35S* promoter along with either a single sgRNA expressed by the *AtU6* promoter or a sgRNA array expressed with the *35S* promoter (Čermák et al., 2017). Additionally, a luciferase reporter driven by either the *35S* or the *CmYLCV* promoter was used as a visual reporter for delivery of the gene editing construct.

### Fast-TrACC

Fast-TrACC involves treating Agrobacterium cultures (GV3101) for 3 days prior to a 2 day co-culture with newly germinated seedlings. The first step is to grow the cultures overnight (8–12 h) in Luria broth (LB) with antibiotics [i.e., kanamycin (50 mg/mL) and gentamycin (50 mg/mL)] at 28°C. Next, cells are harvested by centrifugation and re-suspended to an OD_600_ of 0.3 in AB:MES200 salt solution (17.2 mM K_2_HPO_4_, 8.3 mM NaH_2_PO_4_, 18.7 mM NH_4_Cl, 2 mM KCl, 1.25 mM MgSO_4_, 100 μM CaCl_2_, 10 μM FeSO_4_, 50 mM MES, 2% glucose (w/v), 200 μM acetosyringone, pH 5.5) (Wu et al., 2014) and then grown overnight. The purpose of the AB:MES200 solution is to increase the expression of *vir* genes. The culture is again centrifuged and resuspended to OD_600_ within the range of 0.10–0.18 (typically 0.14) in a 50:50 (v/v) mix of AB:MES200 salt solution and ½ MS liquid plant growth medium (1/2 MS salt supplemented with 0.5% sucrose (w/v), pH 5.5).

Seeds are sterilized using 70% ethanol for 1 min and 50% bleach (v/v) (the hypochlorite concentration of the bleach was 7.4%) for 5 min. They are then rinsed 5 times with sterile water. Seeds are transferred to 6-well plates (~5 seeds per well in 2 mL ½ MS) and maintained in growth chambers (24°C, 16/8 h light/dark cycle). Individual species vary on their germination times (defined as initial cotyledon emergence) in liquid ½ MS: canola seedlings germinate in 2–3 days, *N. benthamiana* seedlings germinate in 3–4 days, tomatoes and potatoes germinate in ~7 days, peppers and eggplant germinate in ~14 days. Two days post germination, ½ MS media is removed and the treated Agrobacterium culture is added. The co-cultured seedlings are incubated for 2 days before being washed free of Agrobacterium using sterile water. The washed seedlings are returned to liquid ½ MS containing the antibiotic timentin at a concentration of 100 μM to effectively counter-select against residual Agrobacterium.

### GFP Imaging and Analysis

Seedlings were assessed for GFP fluorescence using a Nikon Model C-DSD115 stereoscope. Both bright field and GFP fluorescent images were captured from each individual seedling. Images were taken 3 days after removal from co-culture. The software ImageJ was used for GFP image analysis to count cells and determine effectiveness of delivery to each seedling. From the GFP images, the area corresponding to the cotyledons was selected, and background individual puncta were counted using the “Analyze Particles” function.

### Firefly Luciferase Imaging

Seedlings are analyzed for delivery of the T-DNA constructs containing a firefly luciferase reporter through long exposure imaging. Luciferin substrate (5 μL of 50 mM in ddH_2_O stock into 2 mL of ½ MS, final concentration of 125 μM luciferin in ½ MS) is added to the ½ MS liquid culture with the seedlings to produce light. The plate of seedlings is then lightly shaken for 5 min to ensure proper mixing of the luciferin solution. Long-exposure imaging (5.5 min exposure using a UVP Bioimaging Systems EpiChemi^3^ Darkroom) is then performed to capture the luminescence.

### Dual Luciferase Assay

Dual luciferase assays were performed using the Promega Dual Luciferase^®^ Reporter Assay System (Promega Cat. E1910) (Sherf et al., 1996). Treated seedlings were homogenized and resuspended in 1X passive lysis buffer, followed by passive lysis at 70 rpm for 15 min. Lysate was loaded into Grenier 96-well Lumitrac plates for analysis in the Berthold Technologies Centro XS^3^ LB 960 Microplate Luminometer. One hundred microliter of prepared luciferase assay buffer II was injected into a single well, followed by measurement of firefly bioluminescence. Immediately following, 100 μL of prepared Stop & Glo® Buffer was injected into the same single well, and Renilla bioluminescence was measured. Relative Luciferase Units (RLUs) were calculated by taking the firefly:Renilla luminescence ratio, followed by normalization over the negative control. To perform fold change comparisons, the selected promoter’s luminescence ratio was normalized over the luminescence ratio of the other promoters.

### Testing for Editing

Gene editing frequencies in a given set of seedlings were measured by first extracting DNA extracted from selected tissues using CTAB. The isolated DNA was used as a template for PCR amplification of the target locus, and submitted either for next generation sequencing (NGS) (Campbell et al., 2015) or Sanger sequencing. Sanger traces were analyzed by TIDE (Brinkman et al., 2014), which uses software to de-convolute the Sanger peaks to determine editing efficiencies and outcomes. Sanger sequencing trace files from unedited plants were used as controls for the TIDE analysis. Primers for TIDE analysis were standard PCR primers, whereas the primers used for NGS contained 4bp barcodes in the forward and reverse directions, as well as Illumina adapters (Supplementary Table 2). Amplification products were submitted for NGS sequencing using GENEWIZ Amplicon-EZ services (www.genwiz.com). Each pool was de-multiplexed for unique forward and reverse adapters using ea-utils (Aronesty, 2013). Mutations were assessed for each de-multiplexed sample using Cas-Analyzer (Park et al., 2017). Minority read sequences (<10 reads) were considered background.

## RESULTS

### Optimizing Co-culture Conditions for Reliable Delivery of Transgenes to Multiple Species

The AGROBEST method was developed for Arabidopsis to deliver Agrobacterium T-DNAs to seedlings through co-culture (Supplementary Figure 1A) (Wu et al., 2014). When we tested the AGROBEST co-culture conditions (3 day co-culture, OD_600_ = 0.02) in *N. benthamiana*, we found that delivery of a GFP reporter, as measured by fluorescence, was barely detectable (Maher et al., 2020). Further, after a few days, considerable tissue necrosis was observed.

**FIGURE 1 ∣:**
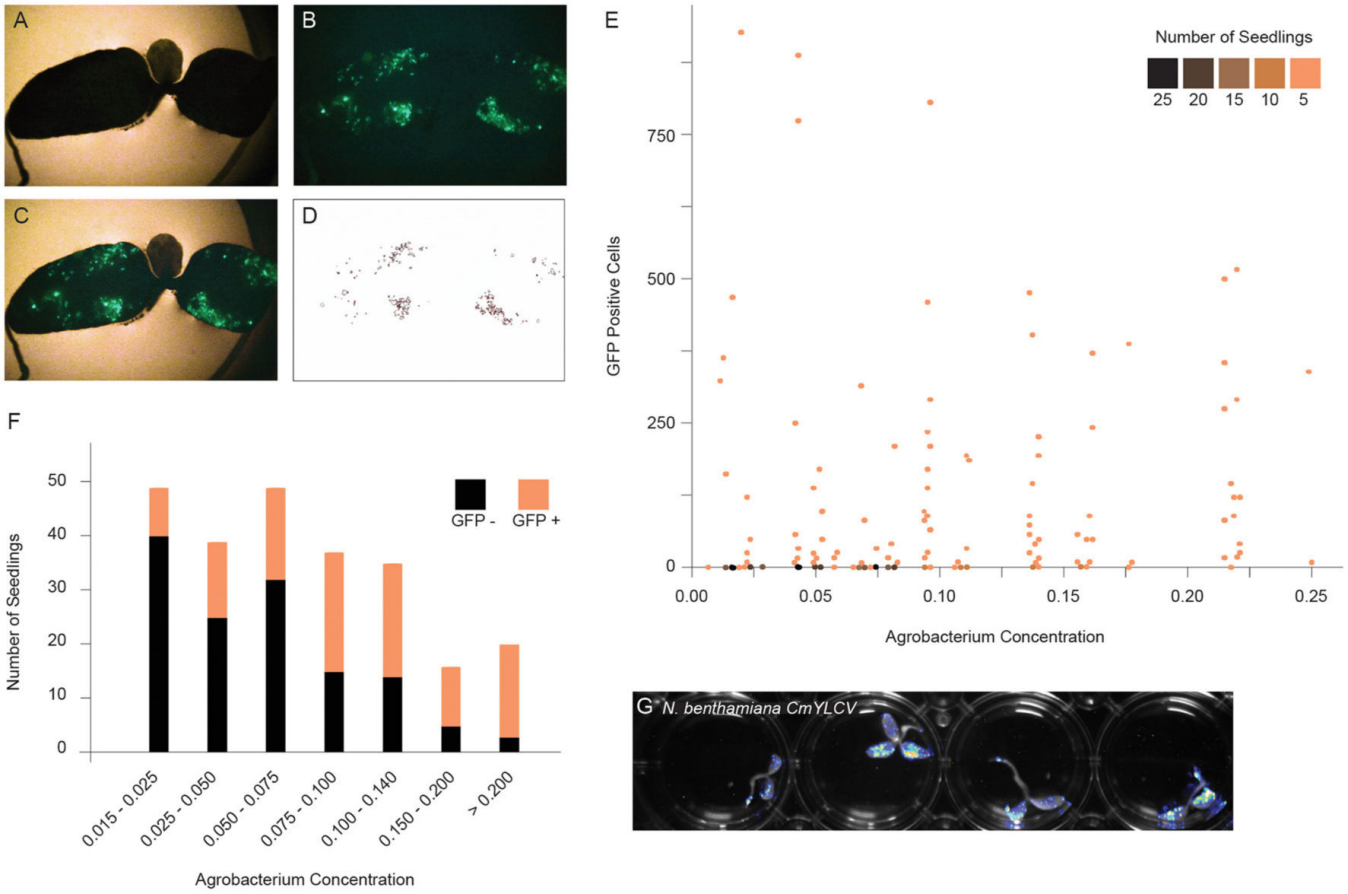
Optimizing fast-TrACC conditions for *N. benthamiana*. To define the optimal co-culture conditions for gene transfer, constructs expressing GFP were delivered to *N. benthamiana* seedlings. After co-culture, seedlings were visualized for the presence of fluorescent signal. Bright field **(A)** and fluorescent images **(B)** were merged **(C)**, and the fluorescent signal was isolated over background **(D)**. Using these images, individual GFP positive sectors were counted. Seedlings were treated across a range of Agrobacterium concentrations, and the number of GFP positive sectors were tracked **(E)**. While seedlings with GFP positive sectors were observed at all bacterial concentrations, the number of negative seedlings was much higher at lower concentrations (**E,F**, black). The Agrobacterium concentration of OD_600_ = 0.09 represents the inflection point where an increasing percentage of seedlings showed fluorescence (**E,F**, orange). While the trend continued beyond OD_600_ = 0.18, there was a subsequent increase in tissue death beyond this concentration. In addition to fluorescent reporters, firefly luciferase can be delivered to *N. benthamiana* seedlings as illustrated here with the *CmYVLC* promoter **(G)**.

To implement a method for delivery of T-DNAs through co-culture to other plant species, we first developed a quantitative assay to measure expression of a GFP reporter in seedlings. The GFP reporter is on a geminiviral replicon to improve expression (Supplementary Figure 1B). Replicons undergo rolling circle replication and thereby significantly increase copy number of transgenes (Baltes et al., 2014). *N. benthamiana* seedlings were co-cultured with varying concentrations of bacteria, and after 2 days, seedlings were photographed under UV light, and GFP fluorescence was quantified by image analysis (Figures 1A-D, Supplementary Figures 1C,D). Although seedlings with GFP positive sectors were observed at all bacteria concentrations, the number of negative seedlings was much higher at lower concentrations (Figure 1E). The Agrobacterium concentration of OD_600_ = 0.09 was the inflection point, above which an increasing percentage of seedlings showed fluorescence (Figure 1F). While the trend of increased fluorescence continued beyond OD_600_ = 0.18, there was a subsequent increase in tissue death beyond this concentration. Ultimately, we selected a 2 day co-culture and an OD_600_ of ~ 0.14. The GFP reporter could be swapped for firefly luciferase, allowing for rapid, whole plate imaging to monitor reagent delivery (Figure 1G).

The Fast-TrACC co-culture conditions used for *N. benthamiana* also worked well for tomato (Figure 5), potato (Figure 6), pepper, eggplant and canola (Supplementary Figures 1F-H). Constructs containing *AtUbi10:luciferase* were delivered to tomato seedlings and expression was observed across the seedlings (Supplementary Figure 2). To assess the transient nature of gene expression using Fast-TrACC, luciferase expression in tomato seedlings was monitored over a 72 h time period. T-DNAs containing either *35S: luciferase* or *AtUBQ10:luciferase* were imaged every 24 h after removal from co-culture. High levels of expression were observed at 24 h, which continually diminished over the next 48 h. Some expression is observed at all time points, which is presumably due to transgene integration. These observations define the timeframe of activity and allow for reagent assessment to be planned accordingly.

### Using Fast-TrACC to Compare Promoter Activity in Different Species

**FIGURE 2 ∣:**
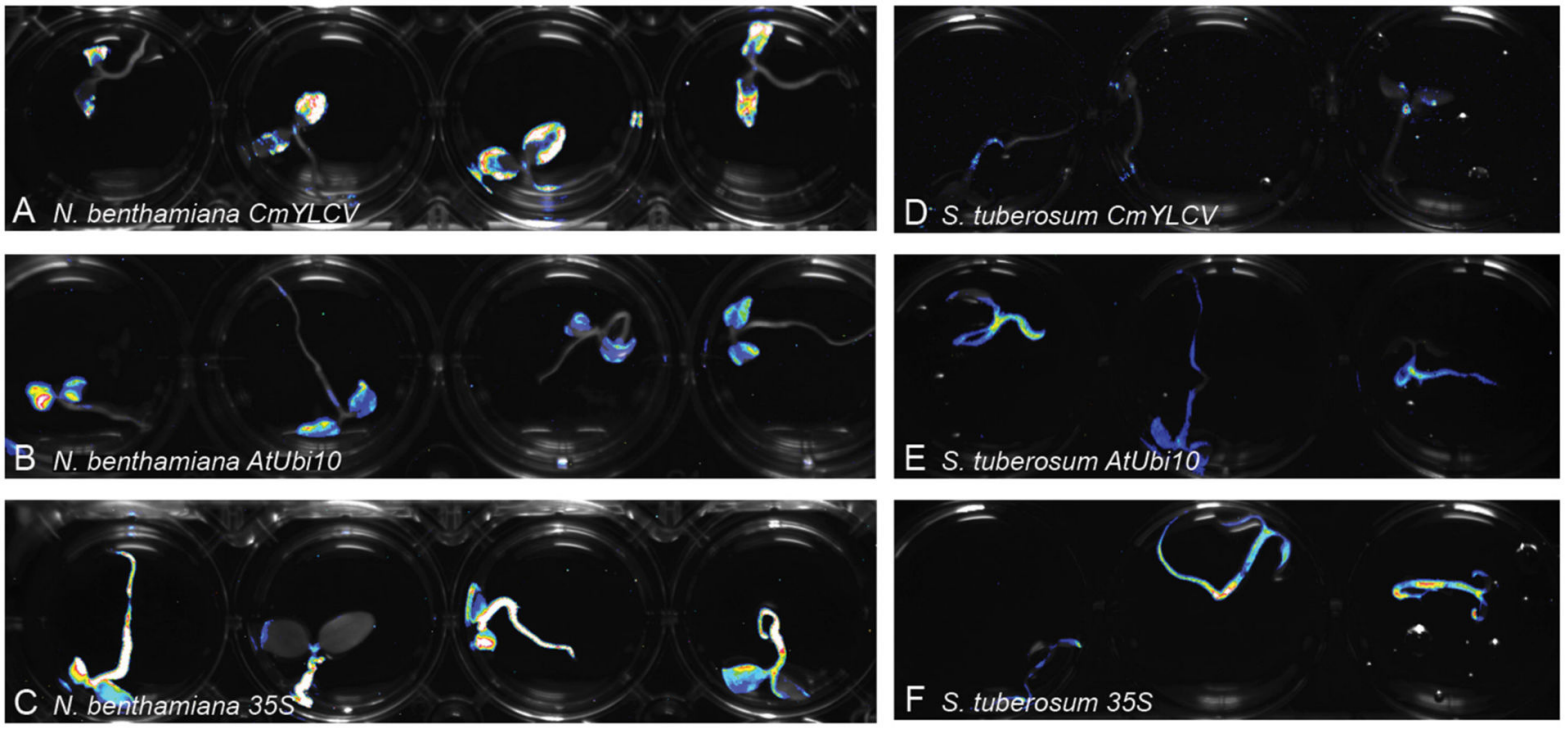
Monitoring differences in promoter expression across species. Firefly luciferase expression was used to compare promoter activity in different species. Constructs encoding luciferase driven by the promoters *CmYLCV* **(A,D)**, *AtUbi10* **(B,E)**, and *35S* **(C,F)** were delivered to *N. benthamiana* **(A–C)** and potato **(D–F)** seedlings using Fast-TrACC. By taking long exposure images after delivery, promoter activity can be compared within a given species or across species. Expression patterns for each of the promoters was distinct. Out of the three tested promoters *CmYLCV* showed the greatest differences between species **(A,D)**. Testing new promoters to drive luciferase allows for their effectiveness to be determined in a species of interest.

We sought to determine if Fast-TrACC can be used to quickly assess promoter activity in different plant species. The *35S*, *AtUbi10* and *CmYLCV* promoters are all known to be effective at driving gene expression in *N. benthamiana* (Engler et al., 2014; Čermák et al., 2017). We fused these promoters to luciferase, and delivered the constructs to potato (Figure 2), pepper, eggplant and canola via Fast-TrACC (Supplementary Figure 3). The *35S* and *AtUbi10* promoters performed well in all species; however, the *CmYLCV* was only functional in eggplant. Fast-TrACC, therefore, can be used to obtain a qualitative readout of promoter activity.

**FIGURE 3 ∣:**
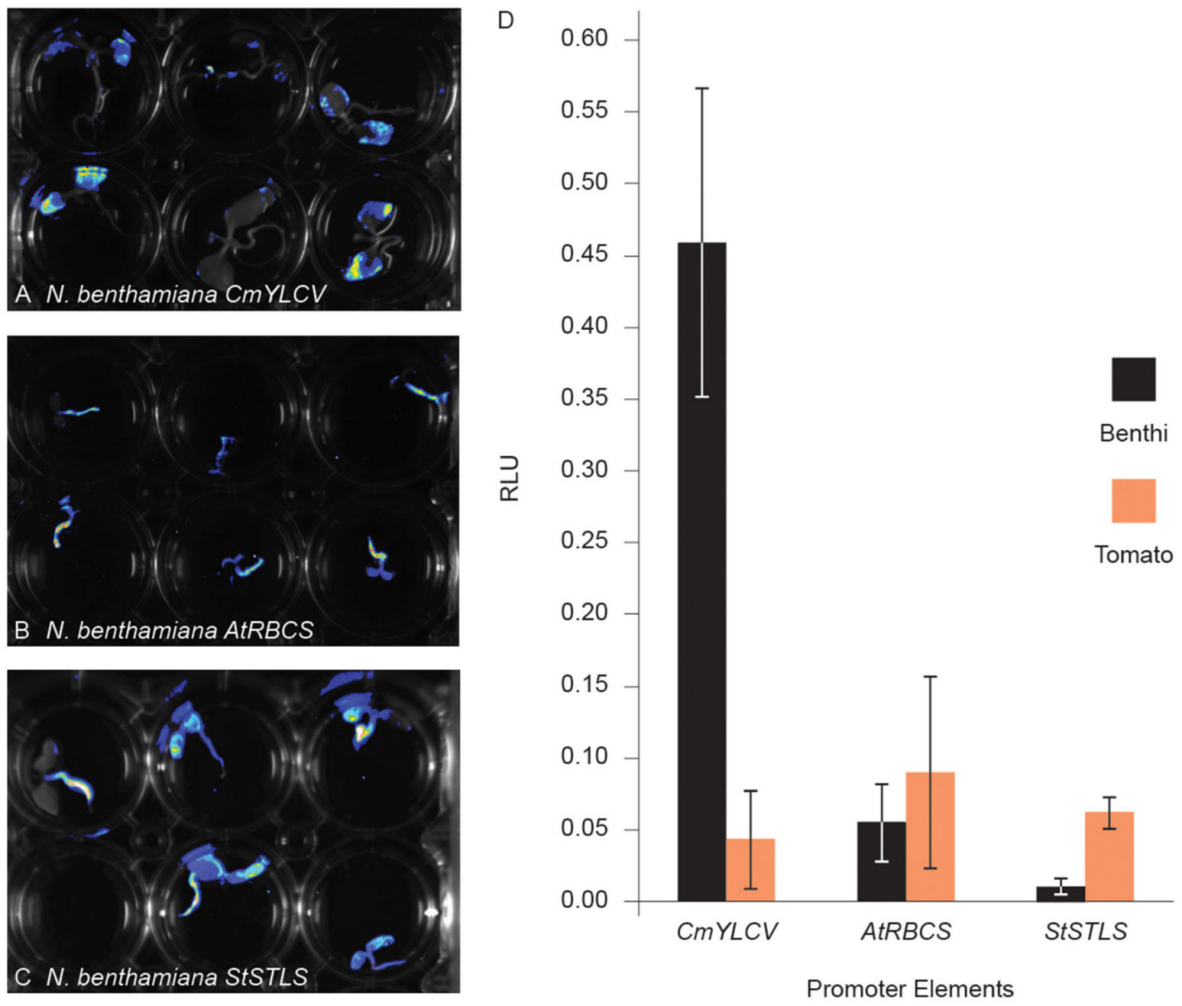
Comparing promoter activity with fast-TrACC using a dual luciferase assay. Activity of three promoters, *CmYLCV* **(A)**, *AtRbcs3B* **(B)**, and *StSTLS* **(C)**, were compared in *N. benthamiana* and tomato. These promoters, driving firefly luciferase, were first delivered to *N. benthamiana* seedlings and qualitatively assessed for activity **(A–C)**. Once promoter activity was confirmed in *N. benthamiana*, T-DNAs with both *35S:Renilla* luciferase and the test promoters driving firefly luciferase were delivered to *N. benthamiana* and tomato seedlings. From seedling-derived lysates, luminescence was recorded for both luciferases. Between the two luminesce values, a relative luciferase unit (RLU) was calculated for the given promoter for direct comparison **(D)**. *CmYLCV* expression was 35-fold higher in *N. benthamiana* when compared to tomato, demonstrating the usefulness of Fast-TrACC for quantitative measurements of promoter activity. Error bars represent ± s.d.

We next sought to determine if quantitative assessments of promoter activity can be achieved using Fast-TrACC. For this, we used a dual luciferase reporter assay (Sherf et al., 1996) to compare the 35S promoter to the *CmYLCV*, Arabidopsis ribulose-1,5-bisphosphate carboxylase/oxygenase small subunit 3B (*AtRbcs3B*), and the potato stem and leaf specific (*StSTLS*) promoters (Figures 3A-C). The three test promoters were fused to firefly luciferase and the *35S* promoter was fused to Renilla luciferase; all constructs were delivered to both *N. benthamiana* and tomato seedlings. While delivery varied, as determined by normalized Renilla luminescence (Supplementary Figure 4), relative expression trends for the test reporters could be discerned. *CmYLCV* yielded much higher expression in *N. benthamiana* than any other promoter in either species (Figure 3D), whereas the *AtRbcs3B* and *StSTLS* promoters were lower in expression and comparable in both species. Specifically, *CmYLCV* was 35-fold higher in expression in *N. benthamiana* relative to tomato, and within *N. benthamiana*, the *CmYLCV* promoter was 27- and 86-fold higher in expression than the *AtRbcs3B* and *StSTLS* promoters, respectively. These results demonstrate that quantitative comparisons can be made between promoter elements across species using Fast-TrACC.

### Using Fast-TrACC to Test Activity of Gene Editing Reagents

**FIGURE 4 ∣:**
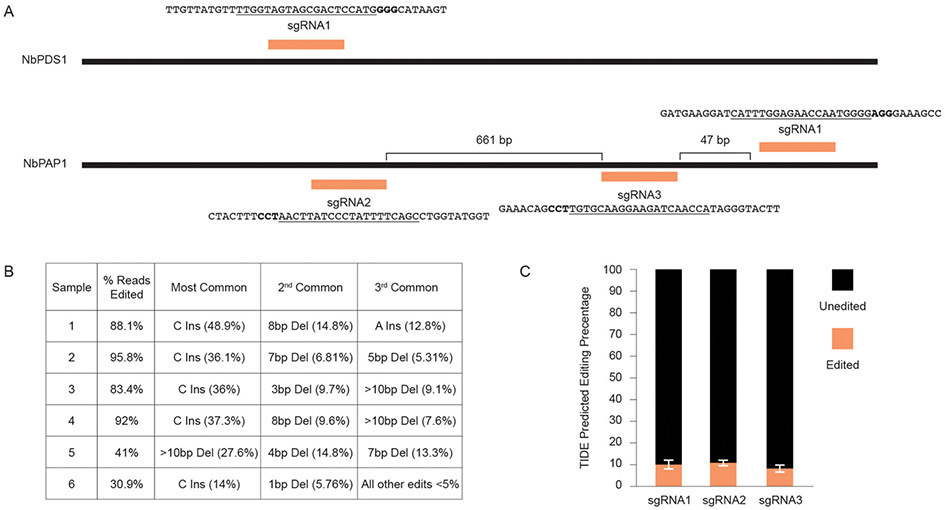
Using fast-TrACC to determine gene editing efficiencies at distinct target sites in *N. benthamiana*. **(A)** Fast-TrACC was used to test a previously characterized sgRNA that targets *NbPDS1* in *N. benthamiana* as well as three untested sgRNAs targeting the *N. benthamiana* locus, *NbPAP1*. The sequences targeted by the sgRNAs are underlined and the PAM sequence is in bold. **(B)** DNA was prepared from six seedlings treated with reagents targeting *NbPDS1;* gene editing frequencies at *NbPDS1* were quantified by NGS. High frequency gene editing was observed in each sample, where editing efficiency is the percentage of total sequencing reads with a gene edit. **(C)** The frequency of editing for the three tested *NbPAP1* sgRNAs was substantially lower than the previously characterized *NbPDS1* sgRNA. sgRNA1 = 10.28% ± 2.18%; sgRNA2 = 11.12% ± 1.47; sgRNA3 = 8.38% ± 1.81.

We next tested whether Fast-TrACC could be used to deliver gene editing reagents to plants to assess their activity. In initial tests, we delivered 35S:Cas9 and a sgRNA targeting the *N. benthamiana phytoene desaturase* (*NbPDS)* locus (Figure 4A). DNA was isolated from each of six treated seedlings, the target site in *NbPDS* was PCR amplified, and the amplicon was subjected to NGS. Each of the six seedlings had gene editing efficiencies ranging from 30 to 95% (Figure 4B). No color change was observed in the seedlings due to loss of *NbPDS*, likely because the cells were photosynthetically competent prior to editing. Additionally, three untested sgRNAs were designed to target the *PURPLE ACID PHOSPHATASE 1* (*NbPAP1)* locus (Figure 4A). Constructs expressing individual sgRNAs were delivered via Fast-TrACC, DNA was isolated from seedlings, and this time editing efficiency was estimated by Sanger-based TIDE analysis. Editing efficiencies were substantially lower for each sgRNA (9–13%, Figure 4C), demonstrating variability in editing across different targets within a species. Since these sgRNAs performed poorly, additional sgRNAs should be tested before attempting to make whole plants with edits in this gene.

**FIGURE 5 ∣:**
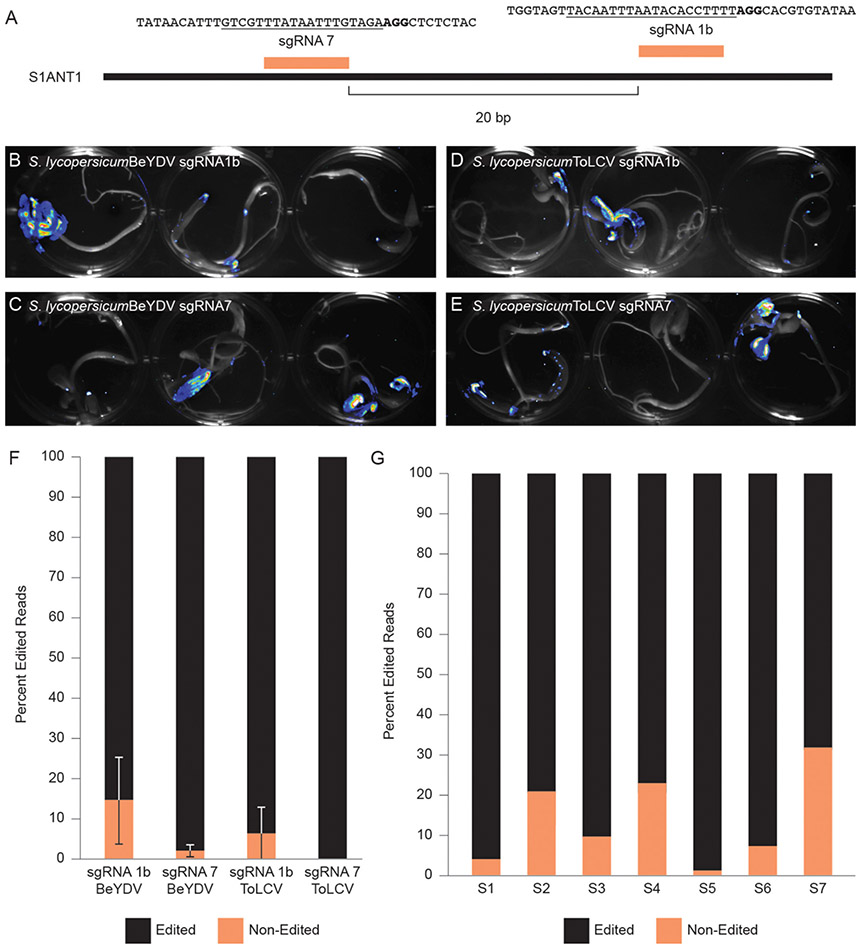
Comparing gene editing efficiencies at a target locus in tomato. **(A)** Two distinct sgRNAs targeting the promoter of *SlANT1* were delivered via Fast-TrACC to tomato seedlings. The sequences targeted by the sgRNAs are underlined and the PAM sequence is in bold. The T-DNAs carried SpCas9, the sgRNAs and a luciferase reporter. These T-DNA sequences contain the required components to form either a BeYDV or ToLCV replicon. Delivery to tomato seedlings of BeYDV replicons with sgRNA1b **(B)** or sgRNA7 **(C)** or ToLCV replicons with sgRNA1b **(D)** or sgRNA7 **(E)** was monitored by luciferase expression and was variable across seedlings. From sectors showing strong luminescence, DNA was collected, and the target site was PCR- amplified and submitted for NGS. Based on the NGS sequencing results, sgRNA1b was more effective at generating edits **(F)** than sgRNA7. Additionally, the ToLCV replicon showed little or no activity **(F)**; Error bars represent ±s.d. When looking at individual seedlings treated with sgRNA1 on a BeYDV replicon, there was noticeable variability in the editing frequency **(G)** likely due to differential construct delivery.

We next determined if we could use Fast-TrACC to test the activity of gene editing reagents outside the *N. benthamiana* model. We delivered to tomato seedlings a constitutive *35S*::Cas9 and one of two sgRNAs (sgRNA1b & sgRNA7) (Figure 5A) that had previously been shown to work at the promoter of the tomato *Anthocyanin 1* (*SlANT1)* (Čermák et al., 2015). These reagents were assembled into T-DNA backbones that produce one of two different viral replicons derived from either Bean Yellow Dwarf Virus (BeYDV) or Tomato Leaf Curl Virus (ToLCV) (Baltes et al., 2014). Also included was a luciferase reporter. As evidenced by the pattern of luminescence (Figures 5B-E), delivery to tomato cotyledons was variable. Cotyledons with luciferase activity were collected, DNA was isolated, and the target site was PCR amplified and assessed for gene editing by NGS. The editing efficiency with sgRNA1b was modest, and editing was barely detectable with sgRNA7 (Figure 5F). When editing efficiencies were assessed at the individual seedling level, considerable variability was observed, likely due to differences in reagent delivery (Figure 5G). Despite the variable delivery, differences in the activity of sgRNAs could be discerned, with sgRNA1b editing at an appreciably higher efficiency on both replicons, whereas sgRNA7 showed little activity and only with the BeYDV replicon (Figure 5F). Thus, Fast-TrACC can be used to assess activity of gene editing reagents to inform decisions regarding sgRNA selection and vector design prior to engaging in lengthy protocols to create plants with heritable gene edits.

**FIGURE 6 ∣:**
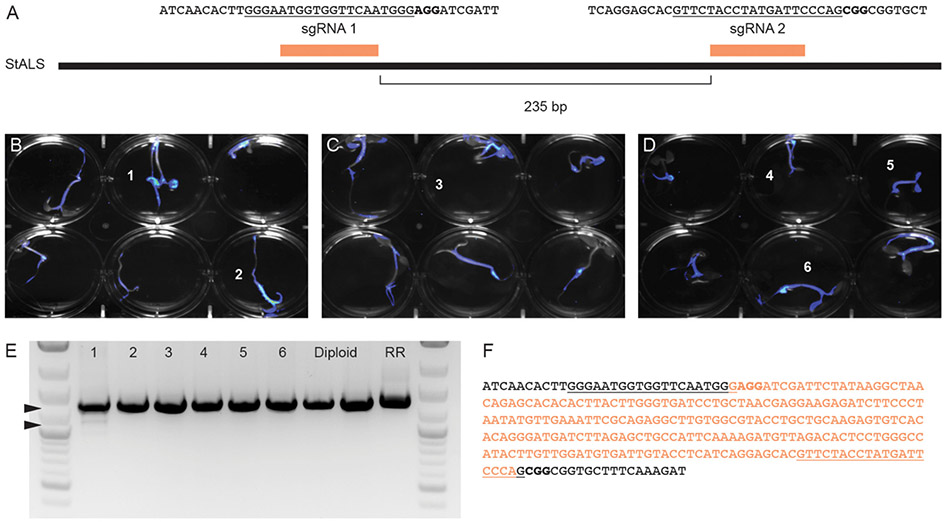
Generating gene edits in potato seedlings. To test gene editing in diploid potato, two previously verified sgRNAs targeting *StALS* **(A)** were cloned into a single T-DNA vector and delivered via Fast-TrACC. The sequences targeted by the sgRNAs are underlined and the PAM sequence is in bold. DNA was isolated from the cotyledons of six seedlings (numbered) with the highest reporter expression **(B–D)**. The *StALS* locus was amplified **(E)** from these DNA samples and a deletion band (lower arrow) was observed in one of the six samples (sample 1, lower arrowhead). This deletion corresponds to the loss of the sequence between the cut sites of the two sgRNAs (**F**, removed sequence in orange, sgRNA sites underlined, PAM sites bolded).

Fast-TrACC was also used to deliver Cas9, sgRNAs and a luciferase reporter to diploid potato seedlings. A previously published pair of sgRNAs targeting the *acetolactate synthase* (*StALS)* locus were used (Figure 6A) (Butler et al., 2015, 2016). The two sgRNAs were delivered together on a tRNA array to allow for individual sgRNAs to be processed from a single transcript. DNA was collected from the cotyledons of six seedlings with prominent luciferase expression (Figures 6B-D, numbered 1–6). The sgRNAs should at some frequency create a 235bp deletion between the sgRNA cut sites, which was observed in one of six tested seedlings (Figure 6E) and verified by DNA sequence analysis (Figure 6F). To this end, we were able to confirm a given set of reagents that generate edits in potato seedlings.

## DISCUSSION

Creating transgenic or gene edited plants is a time-consuming task, often requiring months of effort. Prior to creating such plants, it is valuable to know whether the transgenes are functional or the gene editing reagents are effective in recognizing and cleaving their target sites. There are currently only a handful of ways to transiently test molecular reagents in plant cells, and each has drawbacks. The preparation of protoplasts from plant tissue is time-consuming, requires considerable expertise, and effective protocols are not available for many species. While leaf infiltrations with Agrobacterium are easy to perform, this method is only effective with a handful of plant species. Here we demonstrate that Fast-TrACC provides a quick, low-input, transient delivery method. Although the other methods may end up transforming a higher fraction of treated cells, Fast-TrACC’s scalability and ease of implementation make it an attractive alternative for quickly testing the efficacy of molecular regents.

For expression control elements, such as promoters, we demonstrated that Fast-TrACC could be used for both qualitative and quantitative measurements using luciferase reporters. For example, it was very evident that the CmYLCV promoter had strong species specificity and functioned more effectively in *N. benthamiana* and eggplant than in tomato, potato or canola. Precise gene expression levels were quantified using a dual luciferase assay system. Because expression of test constructs is normalized to a Renilla luciferase cassette on the same T-DNA, the readout is analyzed only in the context of cells that received the construct. While promoters were the primary expression element tested, other expression control elements such as terminators and enhancers could also be tested in a similar fashion.

For gene editing reagents, comparisons could be made between individual sgRNAs targeting the same or different genomic loci. The editing efficiency discrepancies between sgRNAs at distinct genetic loci (as observed in *N. benthamiana*) or at a single locus (as observed in tomato) highlight how variable editing efficiencies can be at different genomic sites and with different sgRNAs and underscores the value in testing gene editing reagents prior to attempting to make gene edited plants. Further, broad species applicability was demonstrated by delivering editing reagents to three distinct species (*N. benthamiana*, tomato and potato). Fast-TrACC thus allows for rapid testing of editing reagents to inform reagent choice.

Fast-TrACC has applications beyond the testing of expression control elements or gene editing reagents. Other molecular reagents could be delivered, such as enzyme expression cassettes or T-DNA-encoded viruses. Previously, we used Fast-TrACC to deliver developmental regulators to whole seedlings, which promoted the formation of *de novo* shoots (Maher et al., 2020). When transgenes or gene editing reagents were co-delivered with the developmental regulators, transgenic or gene edited shoots were induced that transmitted genetic modifications to the next generation. Thus, Fast-TrACC enables a new approach for creating transgenic or gene edited plants.

One of the primary drawbacks to Fast-TrACC is variability in extent of transgene delivery. Agrobacterium is only able to transfer T-DNA to tissues in direct contact with the liquid culture, which leads to certain portions of the seedling being missed, unless completely submerged. This mosaicism has an impact on the functional readout of either promoter activity or gene editing. As mentioned above, the dual luciferase assay addresses the problem of variable delivery, because the Renilla luciferase expression cassette is on the same T-DNA and therefore readouts of expression can be normalized to transformation frequency. For gene editing, efficiencies are underestimated because a fraction of cells never receive the T-DNA. This can be partially compensated for by co-delivering a reporter, and only harvesting and analyzing reporter-positive tissues. Finally, while we demonstrated delivery in a variety of different dicot species, with the exception of canola, all were members of the Solanaceae. Further experimentation will need to be done to determine how broadly Fast-TrACC can be applied across species, and whether, for example, it can be used to transiently transform monocots.

In summary, Fast-TrACC is a simple technique to quickly test molecular reagents for efficacy *in planta*. Although Fast-TrACC has limitations in that gene transfer is often not complete, this drawback is offset by the speed and high-throughput potential of the technique. We expect Fast-TrACC will quickly identify robust molecular reagents that can be applied to help answer lingering questions in the field of plant biology.

## Supplementary Material

Supplementals

## Data Availability

The raw data supporting the conclusions of this article will be made available by the authors, without undue reservation.
