# Supplementary material for: Fast-TrACC: A Rapid Method for Delivering and Testing Gene Editing Reagents in Somatic Plant Cells
Source: Front Genome Ed. 2021 Jan 20;2:621710. doi: 10.3389/fgeed.2020.621710 (PMC8344638; doi:10.3389/fgeed.2020.621710)
Supplement: Supplementary file 1 [file Data_Sheet_1.PDF]

Supplemental Table 1. Constructs delivered by Fast-TrACC.

Construct	MODULE A	MODULE B	MODULE C	MODULE D	VECTOR
pRN62	*35S*:Cas9	*35S*:Non-Targeting gRNAs	*35S*:GFP	--	BeYDV (pCambia T-DNA)
pMM117	*35S*:Luciferase	*AtU6:PDS1* gRNA	--	--	BeYDV (pCambia T-DNA)
pMKV60	--	--	*AtUbi10*:Luciferase	--	BeYDV (pCambia T-DNA)
pMZ94	--	*AtU6:AtWUS* gRNA	*CmYLCV*:Luciferase	--	BeYDV (pCambia T-DNA)
pRN569	--	--	*35S*:Renilla & *CmYLCV*:Firefly	--	pCambia T-DNA
pRN570	--	--	*35S*:Renilla & *AtRbcs*:Firefly	--	pCambia T-DNA
pRN571	--	--	*35S*:Renilla & *StSTLS*:Firefly	--	pCambia T-DNA
pRN221	*35S*:Cas9	*AtU6:PDS1* gRNA	--	--	BeYDV (pCambia T-DNA)
pRN417	*35S*:Cas9	*AtU6:PAP1* gRNA1	*35S*:Luciferase	--	BeYDV (pCambia T-DNA)
pRN418	*35S*:Cas9	*AtU6:PAP1* gRNA2	*35S*:Luciferase	--	BeYDV (pCambia T-DNA)
pRN419	*35S*:Cas9	*AtU6:PAP1* gRNA3	*35S*:Luciferase	--	BeYDV (pCambia T-DNA)
35S:Cas9	*35S*:Cas9	*AtU6:ANT1* Promoter gRNA1b	*CmYLCV*:Luciferase	--	BeYDV (pCambia T-DNA)
pMM181	*35S*:Cas9	*AtU6:ANT1* Promoter gRNA1b	*CmYLCV*:Luciferase	--	BeYDV (pCambia T-DNA)
pMM182	*35S*:Cas9	*AtU6:ANT1* Promoter gRNA1b	*CmYLCV*:Luciferase	--	ToLCV (pCambia T-DNA)
pMM183	*35S*:Cas9	*AtU6:ANT1* Promoter gRNA1b	*CmYLVC*:Luciferase	--	ToLCV (pCambia T-DNA)
pMKV81	*35S*:Cas9	*AtUbi10:ALS* gRNA1 & 2	*AtUbi10*:Luciferase	--	BeYDV (pCambia T-DNA)

Supplemental Table 2. Sequencing Primers.

**Standard/TIDE Primers**		
**Primer Name**	**Description**	**Sequence**
oRN692	*NbPAP1* Rev Primer	GGCACTCGTCGAGCAATTTG
oRN693	*NbPAP1* Fwd Primer	ATTGGCCTGATAGTGGATCA
oMKV108	*StALS* Fwd Amplification Primer	AGAAGGTGAAGTACCCATTGAGTTT
oMKV110	*StALS* Rev Amplification Primer	ATTAAAGGAGGAAATGTGCACCCC
oCS1065	*StALS* Fwd Sequencing Primer	TGGATTAGGAGCAATGGGATTTGG
oCS1243	*StALS* Rev Sequencing Primer	GCCAAAGACCAACGGCAAACTA

**NGS Amplicon Primers**		
**Primer Name**	**Description**	**Sequence**
oRN468	*NbPDS* NGS Fwd Primer (Barcode 1)	ACACTCTTTCCCTACACGACGCTCTTCCGATCT**CAAT**TGCCGTTAATTTGAGAGTCC
oRN469	*NbPDS* NGS Fwd Primer (Barcode 2)	ACACTCTTTCCCTACACGACGCTCTTCCGATCT**CGTT**TGCCGTTAATTTGAGAGTCC
oRN470	*NbPDS* NGS Fwd Primer (Barcode 3)	ACACTCTTTCCCTACACGACGCTCTTCCGATCT**TTCC**TGCCGTTAATTTGAGAGTCC
oRN471	*NbPDS* NGS Fwd Primer (Barcode 4)	ACACTCTTTCCCTACACGACGCTCTTCCGATCT**GTCG**TGCCGTTAATTTGAGAGTCC
oRN472	*NbPDS* NGS Fwd Primer (Barcode 5)	ACACTCTTTCCCTACACGACGCTCTTCCGATCT**GATT**TGCCGTTAATTTGAGAGTCC
oRN473	*NbPDS* NGS Rev Primer (Barcode 1)	GACTGGAGTTCAGACGTGTGCTCTTCCGATCT**TGCT**ATGCTCGTGATCATAAATTC
oRN474	*NbPDS* NGS Rev Primer (Barcode 2)	GACTGGAGTTCAGACGTGTGCTCTTCCGATCT**ATAG**ATGCTCGTGATCATAAATTC
oRN475	*NbPDS* NGS Rev Primer (Barcode 3)	GACTGGAGTTCAGACGTGTGCTCTTCCGATCT**CCCC**ATGCTCGTGATCATAAATTC
oRN476	*NbPDS* NGS Rev Primer (Barcode 4)	GACTGGAGTTCAGACGTGTGCTCTTCCGATCT**TGAA**ATGCTCGTGATCATAAATTC
oRN477	*NbPDS* NGS Rev Primer (Barcode 5)	GACTGGAGTTCAGACGTGTGCTCTTCCGATCT**CGCT**ATGCTCGTGATCATAAATTC
oRN479	Barcode Free *NbPDS* NGS Fwd	ACACTCTTTCCCTACACGACGCTCTTCCGATCTTGCCGTTAATTTGAGAGTCC
oRN480	Barcode Free *NbPDS* NGS Rev	GACTGGAGTTCAGACGTGTGCTCTTCCGATCTATGCTCGTGATCATAAATTC
oRN592	*SlANT1* NGS Fwd Primer	ACACTCTTTCCCTACACGACGCTCTTCCGATCT**GCGA**GCAGGATAGGTACATTGGGA
oRN612	*SlANT1* NGS Rev Primer	GACTGGAGTTCAGACGTGTGCTCTTCCGATCTGTTACCAGCTCTTATGGGAAC

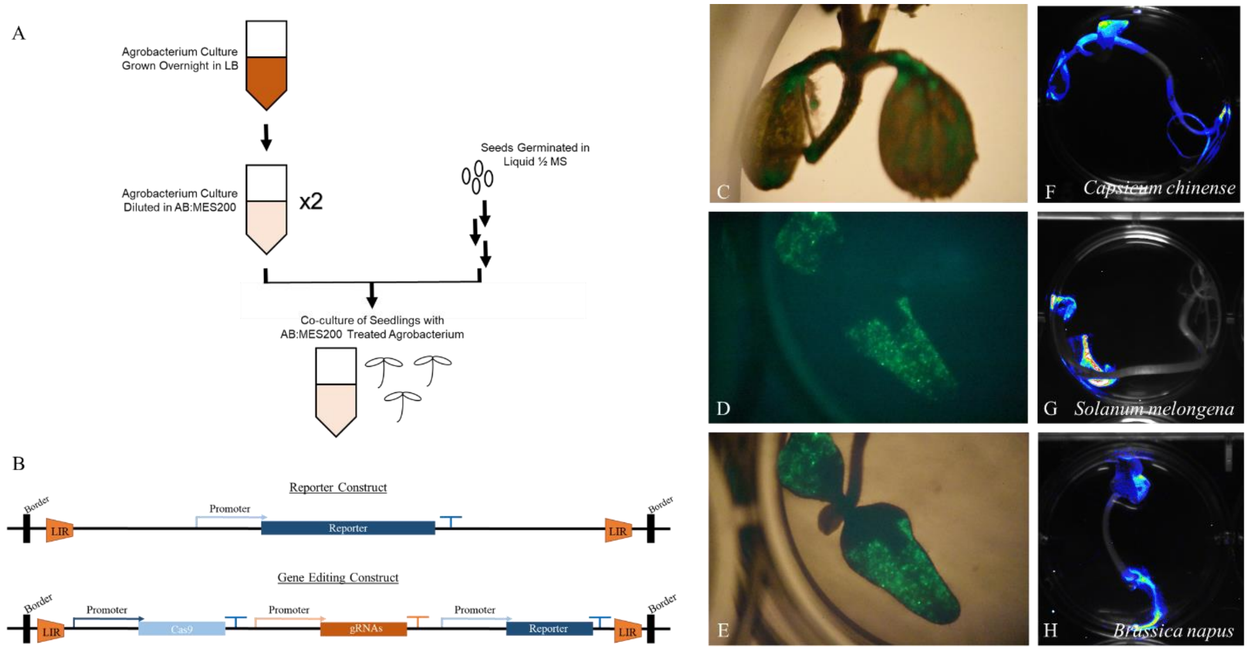

Supplemental Figure 1. Fast-TrACC Delivery of Transgenes. (a) For transgene delivery by Fast-TrACC, Agrobacterium cultures are grown and treated with AB:MES200 salt media to increase *vir* gene expression. (b) T-DNAs are designed to contain geminiviral replicons to increase DNA copy number as well as either a reporter (top) or gene editing reagents and a reporter (bottom). The treated bacterial culture is added to seedlings two days after germination in liquid media. Delivery of reagents, such as GFP (c-e) or luciferase (f-h) to whole seedlings can be performed in multiple species like *Arabidopsis thaliana* (c), *Nicotiana benthamiana* (d-e), habanero pepper (*Capsicum chinense*) (f), eggplant (*Solanum melongena*) (g) or canola (*Brassica napus*) (h).

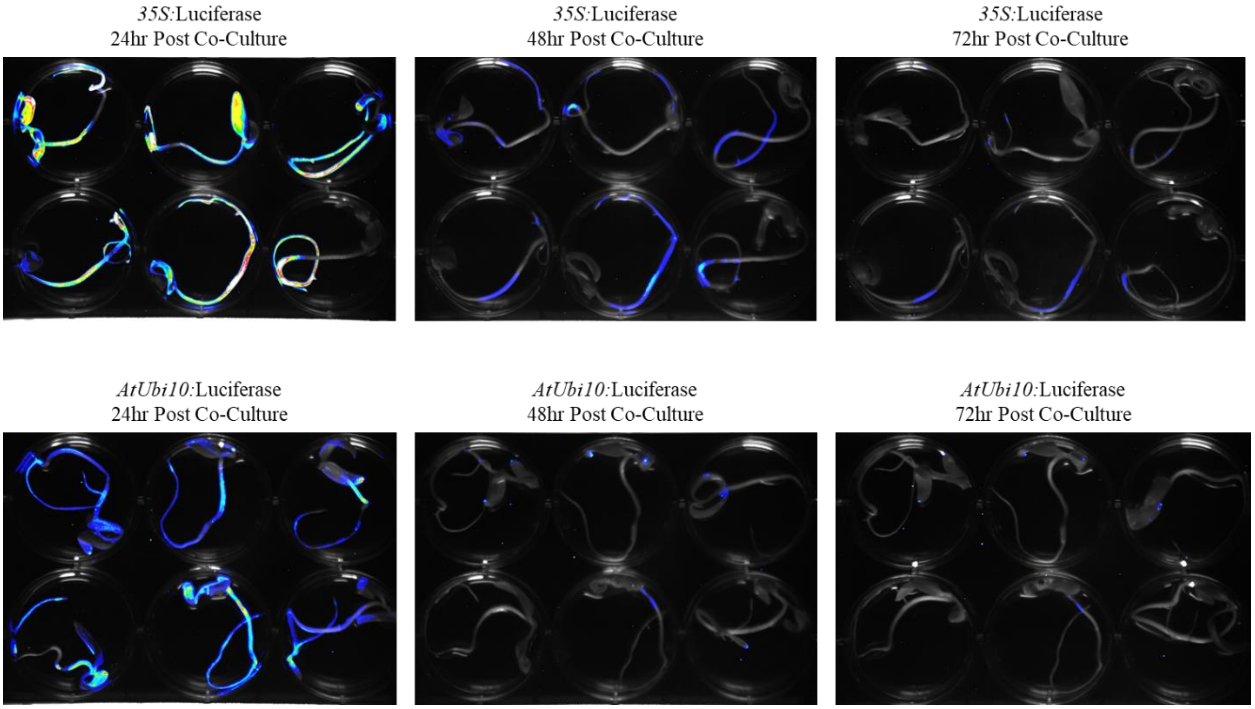

Supplemental Figure 2. Determining the transient nature of Fast-TrACC delivery. Fast-TrACC was used to deliver a firefly luciferase reporter driven by either the *35S* or *AtUbi10* promoter to tomato seedlings. Luminescence serves as a proxy for gene expression from the delivered T-DNA, and the duration of luminescence was recorded. As seen with both *35S* and *AtUbi10*, the luminescence signal is strongest 24 hours after co-culture. This signal drops substantially by 48 hours, and only small sectors of luminescence are observed 72 hours post co-culture. The sectors that remain after 72 hours are likely sites of transgene integration.

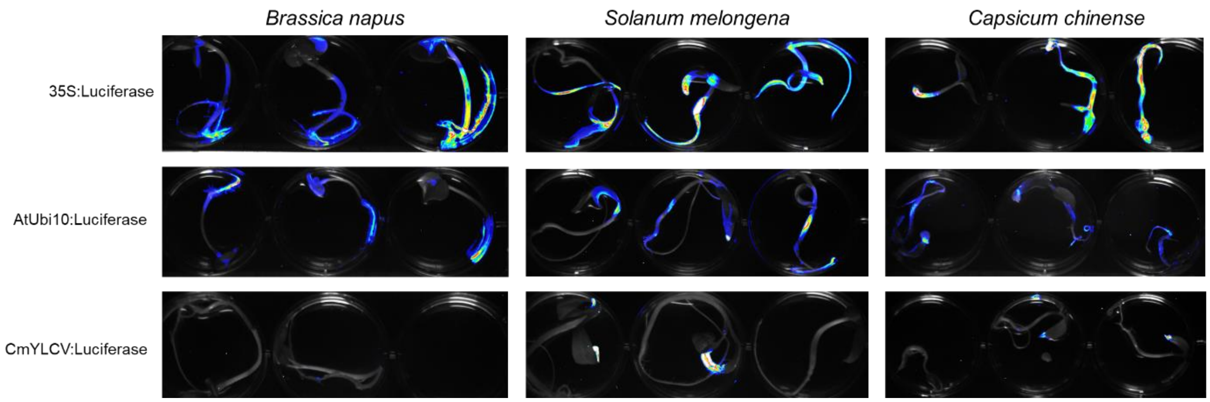

Supplemental Figure 3. Fast-TrACC delivery to various species to determine promoter efficacy. To determine how broadly Fast-TrACC can be applied across species, a firefly luciferase reporter, driven by one of three promoters (*35S*, *AtUbi10*, *CmYLCV*), was delivered to canola (*Brassica napus*), eggplant (*Solanum melongena*), and habanero pepper (*Capsicum chinense*) seedlings. Across the species, *35S:*luciferase manifested strong luminescence, whereas *AtUbi10:*luciferase showed more modest expression. The *CmYLCV* promoter was more species-specific: the strongest expression was observed in eggplant seedlings, but essentially no expression was observed in either the canola or pepper seedlings.

A

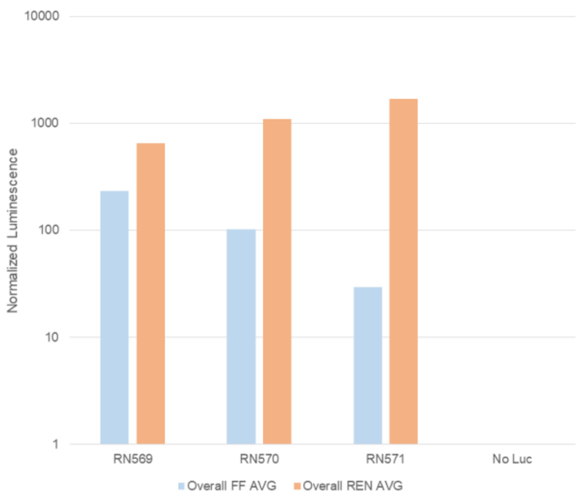

C

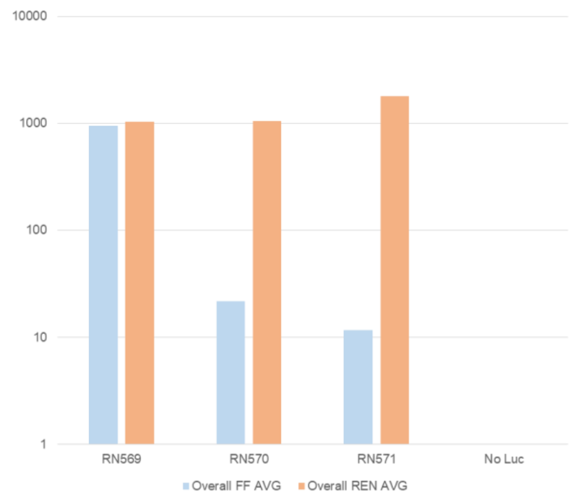

B

Promoter Treatment	Normalized Firefly	Normalized Renilla
RN569 (*CmYLCV*)	234.0363993	646.3191766
RN570 (*AtRbcs*)	102.1347399	1086.951415
RN571 (*StSTLS*)	29.33538311	1700.733103

D

Promoter Treatment	Normalized Firefly	Normalized Renilla
RN569 (*CmYLCV*)	944.1493645	1037.547211
RN570 (*AtRbcs*)	21.5758153	1041.326904
RN571 (*StSTLS*)	11.72038915	1788.561881

Supplemental Figure 4. Normalized luminescence values from a dual luciferase assay. In order to directly compare activity of the *CmYLCV*, *AtRbcs*, and *StSTLS* promoters, a dual luciferase was implemented with firefly and Renilla luciferases. Two independent experiments (a-b, experiment one; c-d, experiment 2) were performed in *N. benthamina*. Raw luminescence values were normalized to the background levels for the no luciferase controls to determine the ratio between the Renilla and firefly enzymes. Variability in the Renilla luciferase luminescence correspond to differential amounts of delivery across the seedlings, whereas the differences in the firefly luciferase values correspond to differing expression levels from the tested promoters. The values from the normalized firefly luciferase measurements are divided by the normalized Renilla luciferase measurements to give a relative luminescence unit (RLU) for comparison.
